# Metabolic consequences of perioperative oral carbohydrates in breast cancer patients — an explorative study

**DOI:** 10.1186/s12885-019-6393-7

**Published:** 2019-12-04

**Authors:** Tone Hoel Lende, Marie Austdal, Tone Frost Bathen, Anne Elin Varhaugvik, Ivar Skaland, Einar Gudlaugsson, Nina G. Egeland, Siri Lunde, Lars A. Akslen, Kristin Jonsdottir, Emiel A. M. Janssen, Håvard Søiland, Jan P. A. Baak

**Affiliations:** 1 Department of Breast & Endocrine Surgery, Stavanger University Hospital, Helse Stavanger HF, P.O. Box 8100, N-4068 Stavanger, Norway; 2 Centre for Cancer Biomarkers CCBIO, Department of Clinical Medicine, Faculty of Medicine and Dentistry, University of Bergen, Jonas Lies vei 87, N-5012 Bergen, Norway; 3 Department of Research, Stavanger University Hospital, Helse Stavanger HF, P.O. Box 8100, N-4068 Stavanger, Norway; 4 Department of Pathology, Stavanger University Hospital, Helse Stavanger HF, P.O. Box 8100, N-4068 Stavanger, Norway; 5 Department of Circulation and Medical Imaging, Norwegian University of Science and Technology, Trondheim, Norway; 6 Department of Pathology, Helse Møre og Romsdal, Ålesund, Norway; 7 Department of Chemistry, Bioscience and Environmental Technology, University of Stavanger, P.O. Box 8600 Forus, N-4036 Stavanger, Norway; 8 Department of Clinical Science, University of Bergen, Jonas Lies vei 87, N-5012 Bergen, Norway; 9 Dr. Med. Jan Baak AS, Risavegen 66, N-4056 Tananger, Norway

**Keywords:** Breast cancer, Carbohydrate load, Proliferation, Insulin, Insulin c-peptide, S-lactate, S-pyruvate, Tumor glutamate, Tumor glutathione, Fasting state, Ketonic bodies, Clinical outcome

## Abstract

**Background:**

The metabolic consequences of preoperative carbohydrate load in breast cancer patients are not known. The present explorative study investigated the systemic and tumor metabolic changes after preoperative per-oral carbohydrate load and their influence on tumor characteristics and survival.

**Methods:**

The study setting was on university hospital level with primary and secondary care functions in south-west Norway. Serum and tumor tissue were sampled from a population-based cohort of 60 patients with operable breast cancer who were randomized to either per-oral carbohydrate load (preOp™; *n* = 25) or standard pre-operative fasting (*n* = 35) before surgery. Magnetic resonance (MR) metabolomics was performed on serum samples from all patients and high-resolution magic angle spinning (HR-MAS) MR analysis on 13 tumor samples available from the fasting group and 16 tumor samples from the carbohydrate group.

**Results:**

Fourteen of 28 metabolites were differently expressed between fasting and carbohydrate groups. Partial least squares discriminant analysis showed a significant difference in the metabolic profile between the fasting and carbohydrate groups, compatible with the endocrine effects of insulin (i.e., increased serum-lactate and pyruvate and decreased ketone bodies and amino acids in the carbohydrate group). Among ER-positive tumors (*n* = 18), glutathione was significantly elevated in the carbohydrate group compared to the fasting group (*p* = 0.002), with a positive correlation between preoperative S-insulin levels and the glutathione content in tumors (*r* = 0.680; *p* = 0.002). In all tumors (*n* = 29), glutamate was increased in tumors with high proliferation (t-test; *p* = 0.009), independent of intervention group. Moreover, there was a positive correlation between tumor size and proliferation markers in the carbohydrate group only. Patients with ER-positive / T2 tumors and high tumor glutathione (≥1.09), high S-lactate (≥56.9), and high S-pyruvate (≥12.5) had inferior clinical outcomes regarding relapse-free survival, breast cancer-specific survival, and overall survival. Moreover, Integrated Pathway Analysis (IPA) in serum revealed activation of five major anabolic metabolic networks contributing to proliferation and growth.

**Conclusions:**

Preoperative carbohydrate load increases systemic levels of lactate and pyruvate and tumor levels of glutathione and glutamate in ER-positive patients. These biological changes may contribute to the inferior clinical outcomes observed in luminal T2 breast cancer patients.

**Trial of registration:**

ClinicalTrials.gov; NCT03886389. Retrospectively registered March 22, 2019.

## Background

Breast cancer is the most common female malignancy and one of the most frequent causes of death among women in the Western world [1]. Breast cancer incidence has more than doubled in the last 50 years, probably due to increased estrogen exposure and a change towards high levels of alimentary carbohydrates and fat [2, 3]. Even though breast cancer originates locally in the breast, circulating tumor cells (CTCs) may spread to the systemic circulation before and during surgery [4] and establish distant micrometastases [5]. These CTCs must thrive and survive attacks from the innate and adaptive immune system. Thus, tumor cells have to establish a favorable metabolism that can produce energy, protection mechanisms, and the necessary biomass to survive the journey from the breast tumor to remote locations, including transformation into dormancy [6]. The luminal breast cancer subtype, which express estrogen receptor (ER) and/or progesterone receptor (PR) in the tumor cells, comprise the largest subgroup, accounting for approximately 75% of all breast cancers. Endocrine resistance in this subtype can creates micrometastases that escape anti-estrogen therapy and can hibernate for many years before they become clinically overt [7]. The molecular features underlying these cellular characteristics are driven by hallmarks of cancer [8], including changes in cellular energetics and metabolism, followed by a vast number of necessary metabolic modifications to strengthen the metabolic needs of breast cancer cells [9]. A well-known cellular characteristic of tumor cells is increased glucose consumption and glycolysis towards lactate despite the presence of oxygen, a feature called ‘the Warburg effect’ [10, 11]. This metabolic switch includes the production of ribose for DNA synthesis and allowing amino acids to be a source for ATP production [12]. Furthermore, the Warburg effect extends to increased choline metabolism for cell membrane synthesis and increased amino acid turnover for protein synthesis [10, 13].

Even though much is known about metabolism in breast cancer cells [14], little is known about the influence of carbohydrate loading in the *early recovery after surgery* (ERAS) program [15] on peri-operative metabolism in the systemic circulation and locally in the breast tumor. We recently conducted a randomized controlled trial (RCT) in which operable breast cancer patients were treated with either two oral loads of enriched carbohydrate solution or a standard fasting procedure comprising free drinking of tap water before surgery [16]. In this study, luminal breast cancer patients, who received oral pre-operative carbohydrates, had a higher tumor proliferation and an adverse survival. The goal of the present paper, using the same patients, was to further explore the metabolic differences in serum and the tumor. Based on our previous findings, we hypothesize that the metabolic changes after carbohydrate loading will correlate with proliferation and outcome in patients with ER positive tumors. Also, we also wanted to study whether such metabolic alterations correlate with other tumor characteristics or translate into differences in clinical outcome.

## Methods

### Ethics statement

This paper is an explorative study based upon a recently published randomized controlled trial (RCT) approved by the Regional Ethics Committee in Western Norway (#2015/1445) and was retrospectively registered at Clinicaltrials.gov (NCT03886389).

### Patients

Details on these patients have been described previously [16]. In short, between 12 May 2009 and 23 June 2010 a population-based cohort of 61 operable breast cancer patients (Stage I and II) were randomized into an intervention group receiving preoperative per-oral carbohydrate loading (*n* = 26) or a control group (*n* = 35) receiving the standard preoperative fasting protocol.

**Fig. 1:**
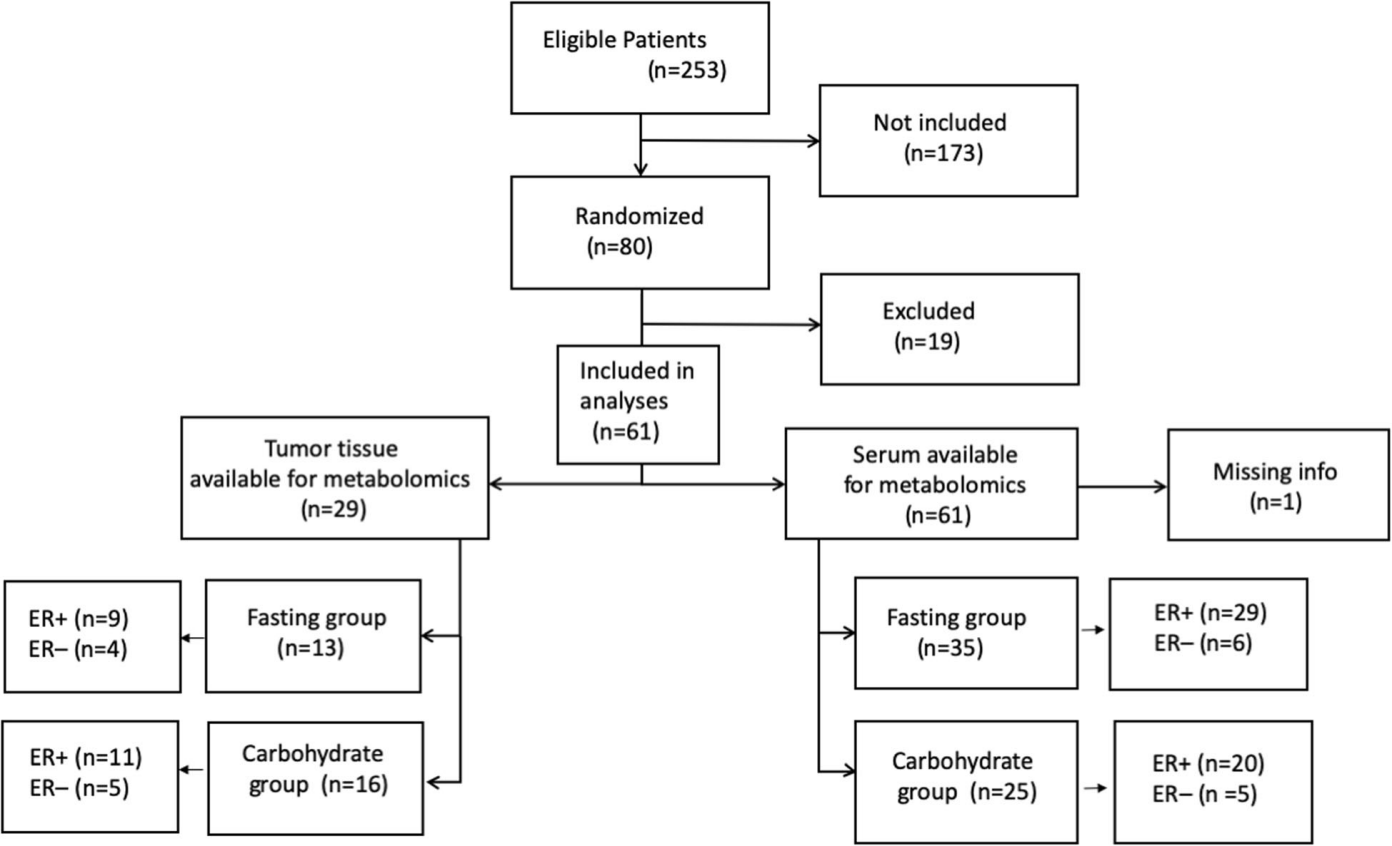
Flowchart of study participants

The patients in the carbohydrate group drank 200 mL pre-Op™ (Nutricia, the Netherlands). This non-carbonated carbohydrate enriched drink contained 100 kCal per bottle containing 4.2 g (2.1%) glucose and 20 g (10%) polysaccharides. A loading dose of two bottles pre-Op™ were given 18 h before surgery (i.e. the evening before surgery) and another 2 bottles were administered 2–4 h before surgery (i.e. the morning of the operation day). In contrast, the control group practiced the standard fasting procedure with free intake of tap water 12–14 h before surgery. From this cohort, patients with available fresh frozen tissue and serum samples were included in the present study (Fig. 1). The patient characteristics are given in Table 1.

**Table 1:** Clinical variables in the randomized groups

	Carbohydrate group (*N*=26)	Fasting group (*N*=35)	Carbohydrate group with tissue (*n*=16)	Fasting group with tissue (*n*=13)
Age				
<55	12 (46%)	16 (46%)	9 (56%)	7 (53%)
≥ 55	14 (54%)	19 (54%)	7 (44%)	6 (46%)
Lymph Node status				
Negative	19 (70%)	25 (71%)	11 (69%)	9 (69%)
Positive	8 (30%)	10 (29%)	5 (31%)	4 (31%)
Tumor size (pT)				
pT1 (<2cm)	16 (61%)	30 (85%)^a^	7 (44%)	9 (69%)
pT2 (≥2cm)	10 (39%)	5 (14%)	9 (57%)	4 (31%)
Grade				
1	4 (15%)	7 (20%)	2 (13%)	2 (15%)
2	10 (37%)	20 (57%)	4 (25%)	7 (53%)
3	13 (48%)	8 (23%)	10 (63%)	4 (31%)
ER status				
Positive	21 (81%)	29 (83%)	11 (69%)	9 (69%)
Negative	5 (19%)	6 (17%)	5 (31%)	4 (31%)
PR status^a^				
Positive	13 (50%)	28 (80%)^b^	7 (44%)	11 (85%)
Negative	13 (50%)	7 (20%)	9 (56%)	2 (15%)
HER2 status				
Negative	23 (88%)	34 (97%)	13 (81%)	12 (92%)
Positive	3 (12%)	1 ( 3%)	3 (19%)	1 (8%)
MAI				
<10	14 (56%)	27 (77%)	6 (38%)	10 (77%)
≥ 10	11 (44%)	8 (23%)	10 (62%)	3 (23%)
PPH3				
<13	14 (56%)	21 (60%)	7 (44%)	6 (46%)
≥ 13	12 (44%)	14 (40%)	9 (56%)	7 (54%)
Ki67				
≥ 15	17 (65%)	17 (50%)	3 (19%)	5 (42%)
<15	9 (35%)	17 (50%)	13 (81%)	7 (58%)
≥ 30	12 (46%)	10 (29%)	6 (38%)	8 (67%)
<30	14 (54%)	24 (71%)	10 (62%)	4 (33%)
TILs				
<10%	24 (92%)	31 (89%)	15 (94%)	13 (100%)
≥10%	2 (8%)	4 (11%)	1 (6%)	0 (0%)
End of follow-up status				
No distant metastasis	22 (85%)	33 (94%)	11 (67%)	11 (85%)
Distant metastasis	4 (15%)	2 (6%)	3 (20%)	1 (7%)

^a^Significantly different between fasting and carbohydrate group (Fisher’s exact test)

^b^*p*=0.052 in tissue subset

### Blood sampling

Blood samples were drawn immediately before surgery. In total three serum gel tubes and one EDTA plasma tube were drawn in this study. One serum gel tube and one EDTA plasma tube were delivered within an hour to the department of medical biochemistry for standard analysis. For metabolomics analyses, two serum gel tubes were centrifuged within one hour at 4 °C, 2500 x g in 10 min. After centrifugation, the serum of the two tubes were mixed and a minimum of 1.1 mL serum were sent for analyses in Haukeland University Hospital, Bergen, Norway, the rest of the serum were stored in 1 mL cryotubes at − 80 °C in the biobank at Stavanger University Hospital, Stavanger, Norway.

### Tumor tissue sampling

Immediately after removal of the surgical specimen from the systemic circulation, it was transported to the Department of Pathology for further sampling. To avoid necrotic areas, cancerous tissue from the invasive front of the tumor (i.e. tumor periphery) was immediately snap-frozen in liquid nitrogen and stored at − 80 °C until assayed for tissue metabolomics. Before HR-MAS analysis, tissues from all of the patients were analyzed consecutively for histopathology and immunohistochemistry as described preciously [16].

### Serum hormone and protein analyses

Serum was transported to the Hormone Laboratory, Haukeland University Hospital, Bergen, Norway. Insulin, insulin c-peptide, insulin growth factor 1 (IGF-1), and insulin growth factor binding protein 3 (IGFBP-3) were measured by the IMMULITE 2000 two-site chemiluminescent immunometric assay (Siemens Medical Solutions Diagnostics).

### Serum metabolomics analyses

A separate aliquot of serum was transported to the MR Core Facility at NTNU, Trondheim, Norway for metabolomics analyses. Thawed samples (100 μL) were mixed with bacteriostatic buffer (100 μL; pH 7.4, 0.075 mM Na_2_HPO_4_, 5 mM NaN_3_, 5 mM TSP), transferred to 3-mm NMR tubes, and stored at 5 °C until analysis (< 15 h). The MR analysis was performed using a Bruker Avance III Ultrashielded Plus 600 MHz spectrometer (Bruker Biospin GmbH, Germany) equipped with a 5 mm QCI Cryoprobe with integrated, cooled pre-amplifiers for ^1^H, ^2^H, and ^13^C. Experiments were fully automated using the SampleJet™ in combination with Icon-NMR in TopSpin 3.1 software (Bruker Biospin). One-dimensional ^1^H Nuclear Overhauser effect spectroscopy (NOESY) and Carr–Purcell–Meiboom–Gill (CPMG) spectra with water presaturation were acquired at 310.15 K. The spectra were Fourier transformed to 128 K after 0.3 Hz exponential line broadening and automatically phased and baseline-corrected. Spectra were further processed in Matlab 2013b (The Mathworks Inc., Natick, MA, USA). The CPMG spectral region between 0.1 and 4.2 ppm was selected for further processing. Chemical shifts were referenced to the left alanine peak at 1.47 ppm. Metabolites were identified based on previous assignment [17, 18]. Twenty-eight metabolites were identified as measurable and their areas calculated by integrating the area under the signal curve.

### Breast tumor tissue metabolomics analyses

In the 29 patients with available tissue, the tumors were larger (45% vs. 9% pT2/3/4, *p* = 0.003), had a higher histological grade (52% vs. 18% grade 3, *p* = 0.022), were more often ER-negative (35% vs. 3%, *p* = 0.002), and had higher proliferation (59% vs. 27% PPH3-positive, *p* = 0.002) than those without tissue. Thus, we had a selection bias of larger, non-luminal and a more proliferative tumors into the present study compared to the original study [16]. Tissue was transported on dry ice to the MR Core Facility at NTNU, Trondheim, Norway, for metabolomics analyses. Tissue samples were prepared frozen on a metal plate bathed in liquid nitrogen to minimize tissue degradation. Biopsies (11.0 ± 2.3 mg) were cut to fit 30 μL disposable inserts (Bruker Biospin Corp, USA) filled with 3 μL D_2_O containing 25 mM formate. The insert containing the frozen sample was placed in a 4-mm diameter zirconium rotor (Bruker, Biospin GmbH, Germany) and kept at − 20 °C until analysis (< 8 h). Spin-echo spectra were acquired on a Bruker Avance DRX600 spectrometer with a ^1^H/^13^C magic angle spinning (MAS) probe with gradient (Bruker Biospin GmbH, Germany) using the following parameters: 5 KHz spin rate, 5 °C probe temperature, 5-min temperature acclimatization before shimming and spectral acquisition, CPMG pulse sequence (cpmgpr1d; Bruker) with 4 s water suppression prior to a 90° excitation pulse, total echo time 77 ms, 256 scans, and spectral width 20 ppm. Spectra were Fourier transformed into 64 K following 0.3 Hz line broadening. Phase correction was performed automatically for each spectrum using TopSpin 3.1.

Spectra were preprocessed in Matlab 2013b as follows [19]. The spectral region between 1.4–4.70 ppm, which contained the majority of the metabolite signals, was selected for further processing. Chemical shifts were referenced to the creatine peak at 3.03 ppm. The spectra were baseline-corrected using asymmetric least squares [20] with parameters λ = 1e7 and *p* = 0.0001, setting the lowest point in each spectrum to zero. Lipid peaks at 4.34–4.27, 4.19–4.14, 2.90–2.7, 2.31–2.18, 2.11–1.92, and 1.68–1.5, and ethanol at 3.67–3.62, were excluded. The resulting spectra were normalized to the total area to correct for differences in sample size and tumor cell content. Metabolite peak assignment was based on previous identification [21]. Twenty metabolites were identified as measurable, and the area under the signal curve in the preprocessed spectra was used to calculate their relative intensities. The metabolite integrals were log10 transformed to satisfy prerequisite assumptions of normality.

### Endpoints

Proliferation differences between the carbohydrate and fasting groups were evaluated by Ki67 (< 15% or ≥ 15 and < 30% or ≥ 30%), mitotic activity index (MAI; < 10 or ≥ 10), and PPH3 (< 13 or ≥ 13). The metabolic response to preoperative oral carbohydrate loading was evaluated in serum (preoperative) by ^1^H NMR and in tumor tissue by HR-MAS MRS.

### Univariate analysis

Metabolite differences between groups were assessed by student T-tests. Correlations between continuous variables were assessed by Pearson correlation. Categorical variables were compared by Chi square tests. *P*-values were considered significant when *p* < 0.05. When multiple variables were compared, the resulting *p*-value tables were corrected for multiple testing by the Benjamini-Hochberg method [22].

### Multivariate analyses (serum and tissue)

Multivariate analyses were performed in R V.3.5 [23] using the package PLS [24] and MetaboAnalyst [25]. Metabolite values were auto-scaled (mean-centered and divided by variance) before multivariate analysis. Principal component analysis (PCA) was performed to evaluate the data sets for outliers. Partial least squares discriminant analysis (PLS-DA) was performed to explore differences in serum and tissue metabolic profiles between categories: carbohydrate loading vs fasting. Partial least squares (PLS) was used to find correlations between the tissue metabolic profile and variables (MAI, PPH3, Ki67, serum (S)-glucose, S-insulin, S-insulin c-peptide, S-IGFR, S-IGFPB3, S-estradiol). Metabolites were evaluated by Variable Importance in Projection (VIP) score. The VIP score is a measure of how important each variable was for creating the discrimination model. It is calculated as a weighted sum of squares of the PLS loadings, where the weights are based on the amount of y-variance explained in each dimension [26]. PLS and PLS-DA classification parameters were evaluated by ‘leave-one-out’ cross validation due to the limited sample numbers. Permutation testing was carried out as an additional model validation; sample classes or responses were shuffled, and the model rebuilt with the same numbers of latent variables as the original model. One thousand permutations were performed, and models were considered significant if the final accuracy (of classification models) or R^2^ (of regression models) were > 95% of the permuted accuracy values (*p* < 0.05).

### Thresholds in survival analyses

Relapse-free survival (RFS) was defined as the time from surgery until a relapse from any site. Breast cancer-specific survival (BCSS) was defined as the time from surgery until death from breast cancer, whereas overall survival (OS) was until death from any cause. Receiver-operator characteristic (ROC) analysis identified optimal thresholds for the various continuous metabolite variables using relapse ‘Yes/No’ as the categorical variable (Table 8 in Appendix). The cut-off values obtained in RFS analysis were also used in the BCSS and OS analyses. In ER-negative patients, none of the explanatory variables with ROC-derived thresholds were significant for analysis of RFS, BCSS, or OS. Therefore, further analyses were limited to ER-positive patients. The ROC-obtained thresholds were confirmed with the minimal *p*-value/maximal Wald-value in a Cox model. In the multivariabel Cox analyses the ‘Forward Wald’ method was primarily used. In cases of an unstable model, a stepwise backward analysis was performed.

### Metabolite set enrichment analysis and ingenuity pathway analysis (IPA)

Serum metabolite levels were uploaded to the Enrichment module of MetaboAnalyst to explore the pathways affected by the carbohydrate intervention. Pathway-associated metabolite sets with sets containing at least two metabolites were used. Pathways with *p*-values ≤0.05 (after FDR correction) were interpreted as significant. Serum metabolites with significantly different expression (*p* = 0.05) and their corresponding fold changes were imported into the Ingenuity Pathway Analysis (IPA) software (Ingenuity, Redwood City, USA) to explore which biological and molecular functions these metabolites were involved in and how these and their direct and indirect target molecules were connected, using the network function in IPA. Additionally, we examined if there were a direct or indirect connection between the top network and seven microRNAs related to tamoxifen resistance from our previous paper [27], using the grow function with a moderate or experimentally observed confidence level.

## Results

### Systemic metabolism

**Fig. 2:**
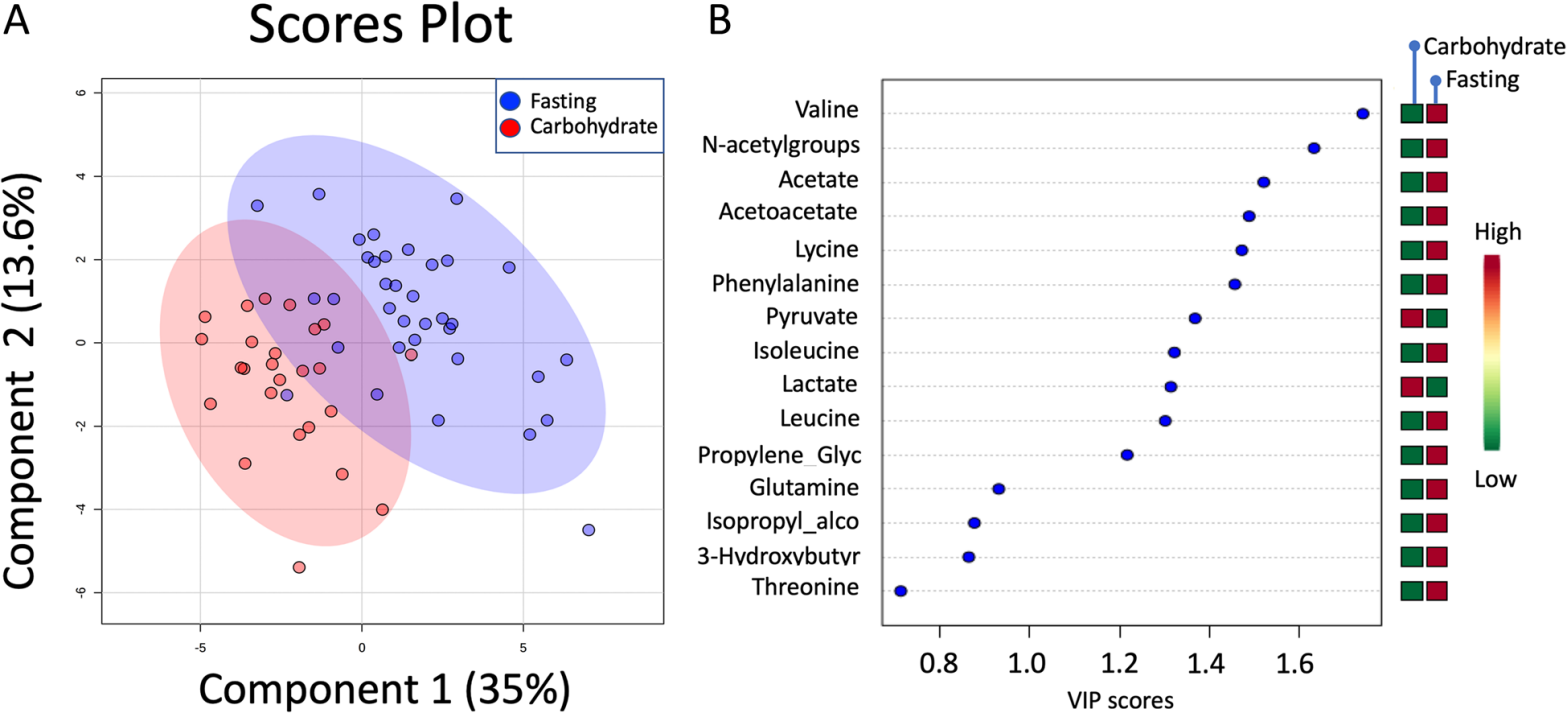
Partial Least Square Discriminant Analysis (PLS-DA) in serum. **a** Scores plot showing serum samples from the fasting group (green) and carbohydrate group (red). The carbohydrate and fasting groups have significantly different metabolic profiles as evidenced by permutation testing. **b** Variable Importance in Projection (VIP) scores showing the top 14 metabolites contributing to differences between the groups. The right column indicates increased (red) or decreased (green) metabolite in the indicated group

The results of the quantification of serum metabolites in the carbohydrate and fasting groups are given in Table 2. Fourteen out of 28 metabolites were significantly altered between the groups. PLS-DA revealed a significant difference in metabolic profiles between the two groups.; (one component, classification accuracy = 0.85; *p* < 0.001; Fig. 2a). The main increased markers were increased serum (S) lactate and S-pyruvate in the carbohydrate group (*p* < 0.0001; Fig. 2a and b). Among the patients in the fasting group, the levels of ketone bodies, such as S-acetate, S-acetoacetate, and S-3-hydroxybutyrate, were increased (Table 2). In addition, we observed increased S-N-acetylated groups, S-leucine, S-valine and S-isoleucine in the fasting group (all *p* < 0.05; Fig. 2b). We found positive correlations between tumor size and S-lactate (*r* = 0.344; *p* = 0.016) and tumor size and S-pyruvate (*r* = 0.370; *p* = 0.009).

**Table 2:** Serum metabolites with *p*-values from t-tests, fasting group versus carbohydrate (CH), for all patients and for the ER positive subset

Metabolite	*p*-value^a^	Fold change	*p*-value^a^ ER+	Fold change ER+
3-Hydroxybutyrate	**0.010**	**-1.06**	**0.010**	**-1.07**
Acetate	**<0.001**	**-1.22**	**<0.001**	**-1.21**
Acetoacetate	**<0.001**	**-1.25**	**<0.001**	**-1.20**
Acetone	0.250	-1.18	0.508	-1.11
Alanine	0.692	1.01	0.544	-1.02
Asparagine	0.237	-1.05	0.376	-1.04
Citrate	0.503	1.03	0.726	1.01
Creatine	0.905	-1.01	0.704	-1.02
Creatinine	0.066	-1.06	0.039	-1.07
Dimethylsulfone	0.319	-1.09	0.154	-1.15
Glucose	0.969	1.00	0.972	1.00
Glutamine	**0.005**	**-1.06**	**0.013**	**-1.07**
Glycerol	0.065	-1.05	0.054	-1.06
Glycoprotein	0.243	-1.06	0.408	-1.05
Isoleucine	**<0.001**	**-1.26**	**0.001**	**-1.22**
Isopropyl alcohol	**0.009**	**-1.12**	0.038	-1.10
Lactate	**<0.001**	**1.36**	**<0.001**	**1.26**
Leucine	**<0.001**	**-1.20**	**0.002**	**-1.17**
Lysine	**<0.001**	**-1.12**	**<0.001**	**-1.11**
Methanol	0.495	-1.04	0.511	-1.04
Methionine	0.052	-1.11	0.062	-1.11
N-acetylated groups	**<0.001**	**-1.15**	**<0.001**	**-1.15**
Phenylalanine	**<0.001**	**-1.12**	**<0.001**	**-1.13**
Proline	0.298	-1.03	0.236	-1.03
Propylene Glycol	**<0.001**	**-1.13**	**0.004**	**-1.10**
Pyruvate	**<0.001**	**1.27**	**<0.001**	**1.23**
Threonine	**0.035**	-1.07	**0.016**	**-1.08**
Valine	**<0.001**	**-1.31**	**<0.001**	**-1.29**

*Abbreviations*: *ER+* Estrogen Receptor positive

^a^Significant at *p* ≤ 0.016 after Benjamini-Hochberg correction for multiple testing

In the carbohydrate group, there was a positive linear correlation between proliferation (Ki-67) and tumor size (*r* = 0.782, *p* = 0.038). When Ki-67, PPH3 and MAI were included in a forward and backward stepwise linear regression MAI was the only independent factor explaining increment in tumor size with a Beta = 0.530 (95%CI, 0.201 to 0.875) *P* = 0.009. In the fasting group, there was no correlation between tumor size and proliferation.’

### Serum glucose and insulin responses

**Fig. 3:**
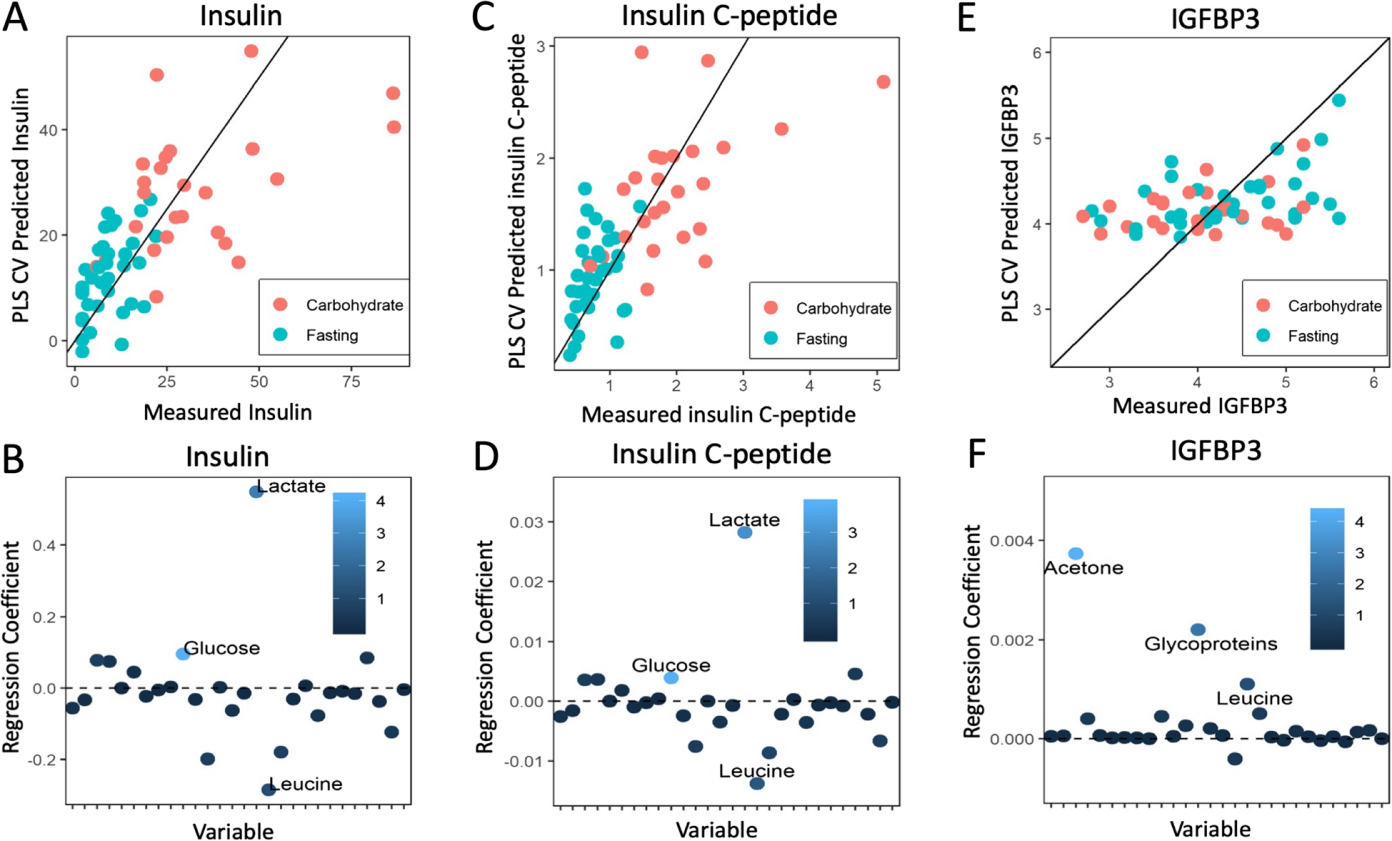
Correlation between serum metabolic profile and serum insulin, insulin C-peptide, and IGFBP3. Samples from carbohydrate-fed patients are shown in red, while samples from fasting patients are shown in blue. Metabolites are colored according to their variable importance in projection (VIP) score and labeled when VIP≥1. **a** Measured insulin vs. predicted insulin levels based on metabolic profile (cross-validated measurements). **b** Metabolites versus regression coefficient for insulin. Increased S-glucose, S-lactate, and decreased S-Leucine are important to prediction of serum insulin from the metabolic profile. **c** Measured insulin C peptide vs. predicted insulin C-peptide levels. **d** Regression weight plot showing metabolites versus the regression coefficient for insulin C-peptide. Increased S-Glucose, S-Lactate, and decreased S-Leucine are important to prediction of serum insulin C-peptide from the metabolic profile. **e** Measured Insulin Growth Factor Binding Protein 3 (IGFBP3) vs. predicted IGFBP3 based on metabolic profile. **f** Regression weight plot showing metabolites versus the regression coefficient for IGFBP3. Increased S-Acetone, S-Glycoproteins, and S-Leucine are important to prediction of serum IGFBP3 from the metabolic profile

The mean fasting glucose and insulin values at admission were 5.4 mmol/L (95% CI 5.1 to 10.0) and 9.4 mIU (95% CI 6.8 to 32.5), respectively (normal ranges: glucose, 4.0 to 6.0 mmol/L; insulin, 6.0 to 27.0 mIU; c-peptide, 0.3 to 2.4 nmol/L). In the carbohydrate group, the mean preoperative insulin value was 35.6 mIU (26.7 to 106 mIU), compared to 9.1 (8.6 to 22 mIU) in the fasting group (student’s t-test *p* < 0.001). For C-peptide, the mean values in the carbohydrate and fasting groups were 2.10 nmol/L and 0.76 nmol/L, respectively (*p* < 0.001). We found significant univariate correlations between the serum concentrations of preoperative insulin (Table 3), Insulin C peptide (Table 9 in Appendix) IGFBP3 (Table 10 in Appendix), but not to IGF1 (Table 11 in Appendix). Multivariate analysis with leave-one-out cross-validation showed significant correlations between the serum metabolic profile and insulin (Cross-validated (CV) (R^2^ = 0.33, *p* < 0.001; Fig. 3a+b), Insulin C-peptide (CV *R*^*2*^ = 0.35, *p* < 0.001; Fig. 3c+d), IGFBP3 (CV *R*^*2*^ = 0.11, *p* < 0.001; Fig. 3e+f), but not IGF-1. For both insulin and insulin C-peptide, the most important metabolites for predictions were increased S-glucose, S-lactate and decreased S-Leucine. For IGFBP3, the most important metabolites were increased S-Acetone, S-Glycoprotein, and S-Leucine. We also found positive correlations between S-lactate and the preoperative increase in S-insulin and S-insulin / c-peptide (*r* = 0.57; *p* < 0.001 and *r* = 0.61; *p* < 0.0001), and between S-pyruvate and the increase in preoperative S-insulin and S-insulin c-peptide (*r* = 0.54; *p* < 0.001 and *r* = 0.60; *p* < 0.001).

**Table 3:** Serum metabolite values correlated to insulin (Pearson’s correlation) for the total study population, and the carbohydrate and fasting groups separately

Metabolite	*R* (All)	*P* (All)*	*R* (CH)	*P* (CH)	*R* (F)	*P* (F)
Lactate	**0.57**	**<0.001**	0.31	0.136	**0.70**	**<0.001**
pyruvate	**0.54**	**<0.001**	0.26	0.203	**0.54**	**0.001**
Acetate	**-0.53**	**<0.001**	-0.40	0.046	-0.22	0.212
N.acetylgroups	**-0.41**	**0.001**	-0.06	0.788	0.10	0.576
Acetoacetate	**-0.34**	**0.008**	-0.04	0.847	0.21	0.221
Valine	-0.31	**0.016**	0.31	0.137	0.28	0.105
Lysine	-0.29	**0.027**	0.01	0.947	**0.43**	**0.010**
Citrate	0.28	**0.029**	0.27	0.192	**0.50**	**0.002**
Isoleucine	-0.28	**0.030**	-0.03	0.881	0.36	**0.035**
Glucose	0.26	**0.043**	0.40	0.047	-0.09	0.622
Propylene_Glycol	-0.24	0.062	0.07	0.748	0.21	0.219
Creatine	-0.23	0.075	-0.39	0.054	-0.18	0.292
Leucine	-0.23	0.079	0.17	0.425	0.25	0.149
Phenylalanine	-0.19	0.149	0.43	0.033	0.24	0.163
Glycerol	-0.19	0.152	-0.12	0.555	0.11	0.541
Alanine	0.15	0.262	0.18	0.386	0.30	0.076
Isopropyl alcohol	-0.12	0.344	0.20	0.331	0.12	0.500
3-Hydroxybutyrate	-0.10	0.442	0.12	0.562	0.36	**0.035**
Methanol	-0.10	0.457	-0.06	0.778	-0.05	0.761
Glutamine	-0.09	0.506	0.30	0.150	0.06	0.737
Creatinine	-0.08	0.543	0.23	0.263	-0.05	0.755
Threonine	-0.08	0.567	0.10	0.627	0.32	0.062
Acetone	0.04	0.780	0.15	0.489	0.39	**0.019**
Proline	-0.04	0.789	0.11	0.593	-0.03	0.880
Glycoproteins	0.02	0.873	0.06	0.787	**0.49**	**0.003**
Asparagine	-0.01	0.923	-0.05	0.818	**0.47**	**0.005**
Methionine	0.01	0.941	0.37	0.067	0.08	0.653
Dimethylsulfone	0.00	0.997	0.17	0.404	0.05	0.777

*Abbreviations*: *CH* Carbohydrate group, *F* Fasting group, *P* Pearson’s correlation *p*-value, *R* Pearson’s correlation *R* value.

*Significant at *p* ≤ 0.043 after Benjamini-Hochberg correction

** Significant at *p* ≤ 0.03 after Benjamini-Hochberg correction

*** Significant at *p* ≤ 0.035 after Benjamini-Hochberg correction

### Tumor metabolism

**Fig. 4:**
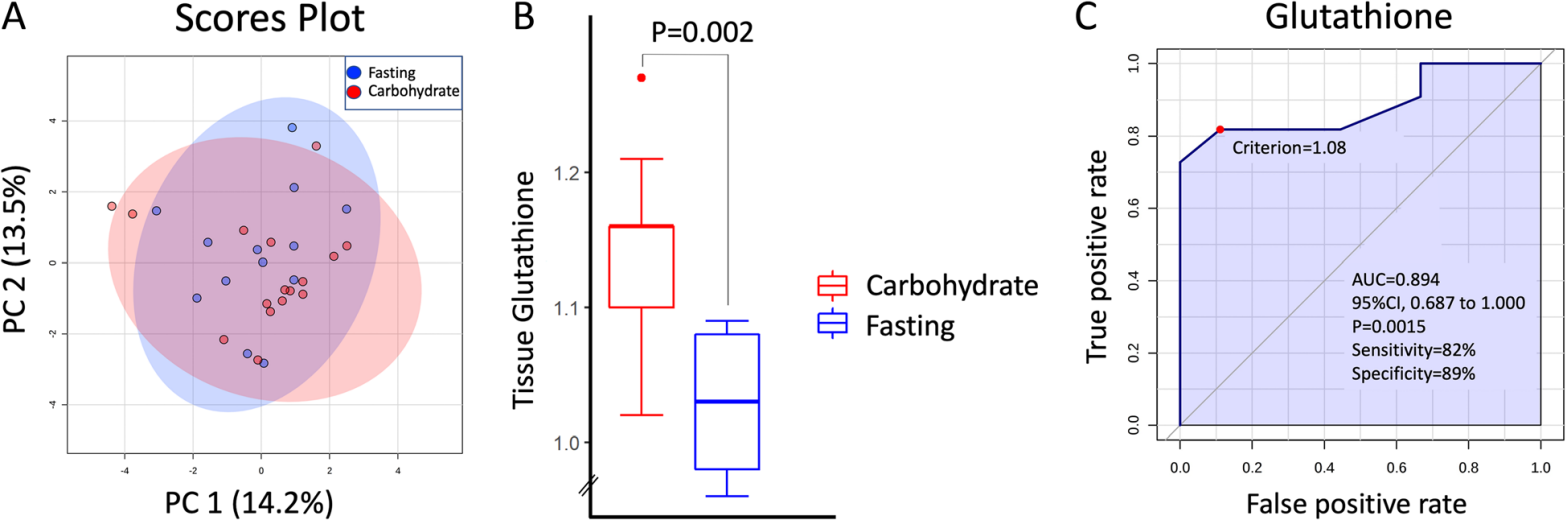
**a** Principal Component Analysis (PCA) of tumor metabolites. No grouping of fasting vs carbohydrate groups observed. **b** Glutathione levels in ER positive tumors. **c** ROC curve for classification into carbohydrate or fasting group by glutathione concentration in ER-positive tumors. AUC= 0.894; 95%CI=0.0.687-1.000, P=0.002

Metabolites included in the analysis are presented in Table 4. PLS-DA did not result in a significant model discriminating between fasting and carbohydrate-fed patients, and no metabolites were significantly different in univariate testing when all tumors were analyzed (Fig. 4a). However, for ER-positive tumors (*n* = 18), glutathione was significantly elevated in the carbohydrate group compared to the fasting group (*p* = 0.002; Fig. 4b), even after adjusting for tumor size. In the ROC analysis, we found an area under the curve (AUC) of 0.894 (95%CI = 0.687–1.000, *p* = 0.0015) for glutathione in discriminating between fasting and carbohydrate-fed patients with ER-positive tumors (Fig. 4c). The difference was also significant in the ER-positive tumors with low proliferation (MAI < 10; *n* = 7). Moreover, we found a positive correlation between preoperative S-insulin levels and the glutathione content in tumor tissue (*r* = 0.680; *p* = 0.002). Furthermore, we observed a higher level of tissue glutamate in tumors with a high proliferation as measured by Ki67</≥ 15% (*p* = 0.004). This association remained significant when adjusted for intervention group using a general linear model with intervention status as fixed factor, Ki67</≥ 15% as random factor, and tissue Glutamate as dependent variable (*p* = 0.009). Also, choline (*p* = 0.002) and phosphoetanolamine (*p* = 0.019) were increased in T2 tumors compared to T1 tumors.

**Table 4:** Tumor metabolites with fold changes and t-test p-values in carbohydrate vs fasting groups

Tumor metabolite	*P* (All)^a^	FC (All)	*P* (ER+)^b^	FC (ER+)
Acetate	0.844	-1.030	0.620	-1.095
Alanine	0.322	1.038	0.163	1.067
Ascorbate	0.300	-1.099	0.991	-1.001
Aspartate	0.385	1.100	0.545	1.088
Choline	0.136	1.056	0.547	1.027
Creatine	0.418	-1.062	0.558	-1.051
Glucose	0.495	-1.151	0.500	-1.201
Glutamate	0.172	1.047	0.146	1.055
Glutamine	0.955	1.003	0.816	-1.015
Glutathione	**0.006**	**1.082**	**0.002**	**1.103**
Glycerophosphocholine	0.712	-1.018	0.762	-1.018
Glycine	0.186	1.063	0.162	1.090
Lactate	0.862	1.006	0.922	1.004
Leucine	1.000	1.000	0.947	-1.004
Myoinositol	0.445	-1.038	0.768	-1.018
Phosphocholine	0.517	1.027	0.291	1.051
Phosphoethanolamine	0.211	1.050	0.544	1.031
Scylloinositol	0.926	-1.007	0.565	1.060
Succinate	0.788	1.022	0.503	1.067
Taurine	0.982	1.001	0.902	1.004

*Abbreviations*: *ER+* Estrogen receptor positive, *FC* Fold change, *P* T-test *p*-value

^a^Significant at *p* ≤ 0.001 after Benjamini-Hochberg correction

^b^Significant at *p* ≤0.030 after Benjamini-Hochberg correction

### Survival analysis

**Fig. 5:**
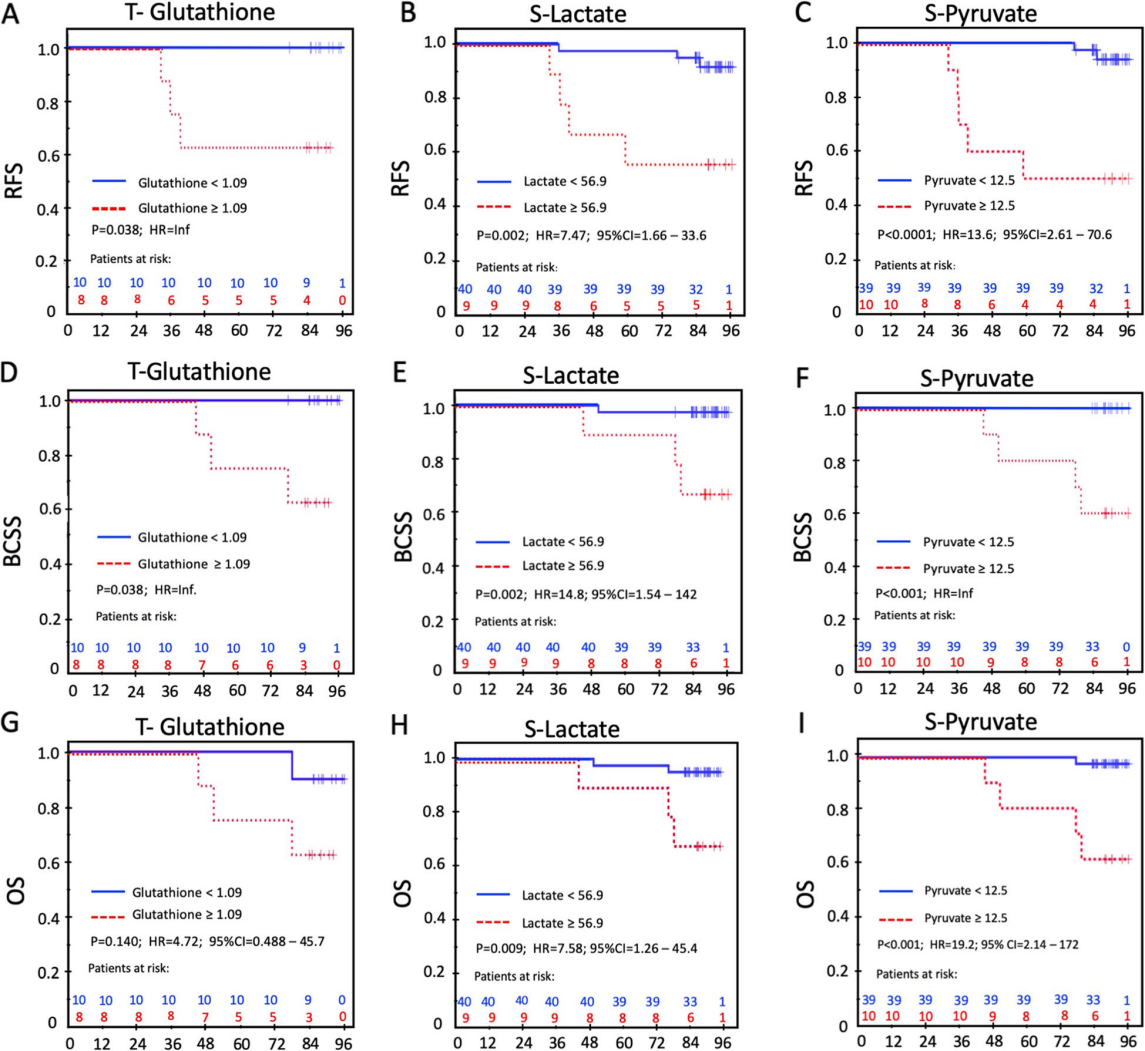
Survival analyses for Tumor-Glutathione, Serum-lactate and Serum-pyruvate. **a-c** Relapse Free Survival (RFS); **d-f** Breast Cancer Survival (BCSS); **g-i** Overall Survival (OS)

First, we used S-lactate, S-pyruvate, and tissue (T) glutathione as continuous variables in a univariate Cox model for RFS, BCSS and OS. Both S-pyruvate and S-lactate, but not T-glutathione reached significance with a hazard ratio (HR) for RFS of 1.53 (95% CI, 1.11 to 2.11; *p* = 0.009) and 1.08 (95% CI, 1.01 to 1.17; *p* = 0.029), respectively. For BCSS the HR for the continuous variables of S-pyruvate and S-Lactate were 1.85 (95%CI, 1.15 to 2.97; *p* = 0.011) and 1.13 (95%CI, 1.01 1.26; *p* = 0.028) respectively. The corresponding observations for OS were 1.63 (95%CI, 1.11 to 2.40; *p* = 0.014) for lactate and 1.10 (95%CI, 1.002 to 1.20; *p* = 0.045) for pyruvate. Thereafter, the following independent variables were dichotomized according to the optimal ROC-derived thresholds: S-lactate, S-pyruvate, preoperative S-insulin, preoperative S-insulin-c-peptide, and tissue glutathione. In addition, the well-established prognostic factors tumor size, nodal status, histological grade, MAI 10, Ki-67-30 and PPH3–13 were deemed clinically relevant and included as explanatory variables in the multivariable analyses. The results of the univariate RFS, BCSS, and OS analyses are given in Tables 5, 6 and 7, respectively. Patients with a high glutathione content in the tumor (≥1.09) had a 37% risk of experiencing a relapse and 37% risk of dying of breast cancer compared to no relapses and no deaths in patients with a low glutathione content in the tumor (both comparisons: *p* = 0.038; HR = Inf.; Fig. 5a and d). Patients with high S-lactate (≥56.9) had RFS of 71% compared to 97% for those with lower S-lactate (*p* = 0.002, HR = 7.47; 95% CI 1.66–33.6; Fig. 5b). Patients with S-pyruvate ≥12.5 had an adverse RFS of 50% compared to 95% for the patients with S-pyruvate < 12.5 (*p* < 0.0001; HR = 13.6; 95% CI 2.61–70.6; Fig. 5c). The same pattern was observed in the BCSS and OS analyses for these three prognostic variables (Fig. 5e-i). Notably, only one contralateral relapse occurred in the fasting group – all others were in the carbohydrate group. Even though the relapses were restricted to patients with T2 tumors, tumor category was not an independent prognostic factor in the multivariable analyses. In the multivariable analysis for RFS, S-pyruvate was the only factor left in the final model (HR = 12.8; 95% CI, 2.47 to 66.8), and only S-lactate remained in the final multivariable model for BCSS (HR = 14.8; 95% CI 1.54 to 142). Furthermore, S-pyruvate was the sole factor to reach significance in the multivariable model of the OS analysis (HR = 18.2; 95% CI 2.03 to 164).

**Table 5:** Univariate analysis of Relapse Free Survival in ER+ patients

Variable	Events / At risk	% Survival	*P*	HR	95% CI
Fasting / Carbohydrate					
Fasting	1/29	97			
Carbohydrate	6/21	71	0.012	9.34	1.12 –77.7
S-Pyruvate^a^					
< 12.5	2/39	95			
≥ 12.5	5/10	50	<0.0001	13.59	2.61– 70.6
S-Lactate^a^					
< 56.9	3/40	93			
≥ 56.9	4/9	56	0.002	7.47	1.66 – 33.6
S-Preoperative Insulin					
< 18.3 I.U.	1/29	97			
≥ 18.3 I.U.	6/21	71	0.012	9.34	1.12 – 77.7
S-Preoperative C-peptide					
< 1.22 nM	1/29	97			
≥1.22 nM	6/21	71	0.011	9.51	1.14-79.0
Tumor Glutathione					
< 1.09	0/10	100			
≥ 1.09	3/8	63	0.038	Inf.	
Tumor size					
T1	3/40	93			
T2	4/10	60	0.003	7.09	1.57-31.9
Nodal status					
N0	3/33	91			
N+	4/17	73	0.160	2.80	0.625-12.6
Grade					
1	0/11	100			
2+3	7/39	82	0.136	31.1	0.019 – 50547
MAI^a^					
<10	4/39	90			
≥10	3/10	70	0.092	3.38	0.751–15.2
Ki67^a^					
<30%	3/37	92			
≥30%	4/12	67	0.023	4.84	1.08 – 21.8
PPH3					
<13	3/35	91			
≥13	4/15	73	0.116	3.13	0.699-14.0

^a^Missing information on one patient in the ER+ group leading to *n*=49 patients analyzed for this variable

**Table 6:** Univariate analysis of Breast Cancer Specific Survival in ER+ patients

Variable	Events / At risk	% Survival	*P*	HR	95% CI
Fasting /Carbohydrate					
Fasting	0/29	100			
Carbohydrate	4/21	81	0.015	Inf.	
S-Pyruvate					
< 12.5	0/40	100			
≥ 12.5	4/10	60	<0. 0001	Inf.	
S-Lactate^a^					
< 56.9	1/40	98			
≥ 56.9	3/9	67	0.002	14.8	1.53-142
S-Preoperative Insulin					
< 18.3 I.U.	0/29	100			
≥ 18.3 I.U.	4/21	81	0.015	Inf.	
S-Preoperative C-peptide					
< 1.22 nM	0/29	100			
≥1.22 nM	4/21	81	0.015	103	0.025-429676
Tumor Glutathione					
< 1.09	0/10	100			
≥ 1.09	3/8	63	0.038	Inf.	
Tumor size					
T1	0/40	100			
T2	4/10	60	<0.0001	Inf.	
Nodal status					
N0	1/33	97			
N+	3/17	82	0.080	5.92	0.615 – 56.9
Grade					
1	0/11	100			
2+3	4/39	90	0.277	30.1	Inf.
MAI^a^					
<10	2/39	95			
≥10	2/10	80	0.124	4.12	0.580- 29.3
Ki67^a^					
<30%	1/37	97			
≥30%	3/12	75	0.014	9.91	1.03-95.3
PPH3					
<13	2/35	94			
≥13	2/15	87	0.399	2.27	0.320 – 16.1

^a^Missing information on one patient in the ER+ group leading to *n*=49 patients analyzed for this variable

**Table 7:** Univariate analysis of Overall Survival in ER+ patients

Variable	Events /At risk	% survival	*P*	HR	95% CI
Carbo/Faste					
Faste	1/29	97			
Carbohydrate	4/21	81	0.068	6.02	0.675–53.8
S-Pyruvat^a^					
< 12.5	1/39	97			
≥ 12.5	4/10	60	<0.0001	19.2	2.14–172
S-Lactate^a^					
< 56.9	2/40	95			
≥ 56.9	3/9	67	0.009	7.58	1.26–45.4
S-Preop Insulin					
< 18.3 I.U.	1/29	97			
≥ 18.3 I.U.	4/21	81	0.068	6.016	0.672–53.9
S-Preoperative C-peptide					
< 1.22 nM	1/29	97			
≥1.22 nM	4/21	81	0.068	6.02	0.672–53.9
Tissue Glutathione					
≤1.0855	1/10	90			
>1.0855	3/8	63	0.140	4.72	0.488–45.7
Tumor size					
T1	1/40	98			
T2	4/10	60	< 0.0001	19.2	2.20 –176
Nodal status					
N0	2/33	94			
N+	3/17	82	0.205	3.01	0.502–18.0
Grade					
1	0/11	100			
2+3	5/39	87	0.222	30.2	0.004–223736
MAI^a^					
<10	3/39	92			
≥10	2/10	80	0.235	2.83	0.471–16.9
Ki67^a^					
<30%	2/37	95			
≥30%	3/12	75	0.049	5.040	0.842–30.2
PPH3					
<13	3/35	91			
≥13	2/15	87	0.641	1.53	0.255–9.13

^a^Missing information on one patient in the ER+ group leading to *n*=49 patients analyzed for this variable

### Pathway analyses

**Fig. 6:**
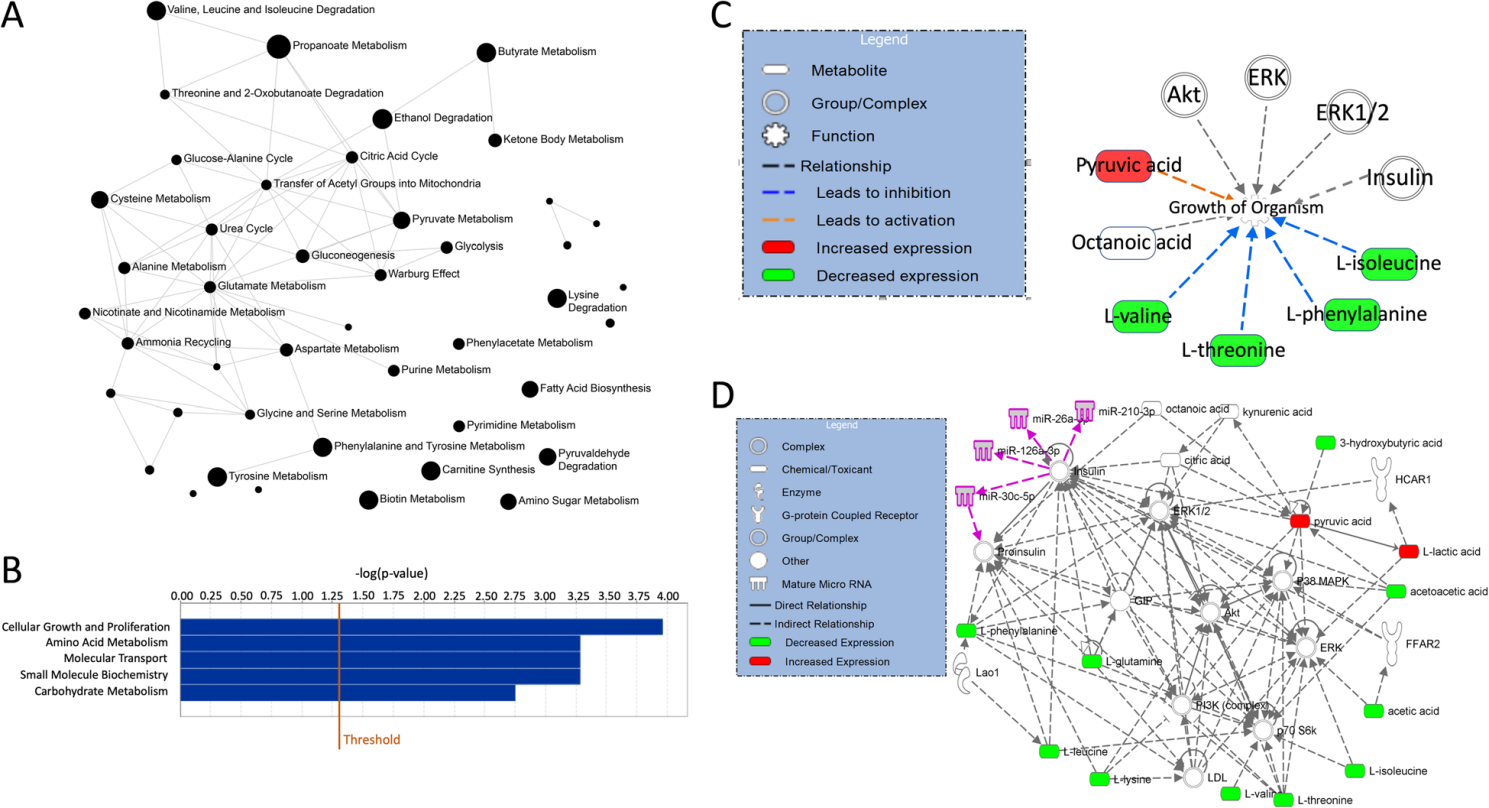
Pathway analyses in serum metabolites. **a** Metabolite Set Enrichment Analysis of serum metabolism. Significantly enriched pathways are annotated in the pathway network. The circle size denotes significance of the pathway, and lines denote at least 25% shared metabolites in the pathways. **b** Ingenuity pathway analysis (IPA) bar chart showing the top 5 functions enriched in the dataset. **c** IPA pathway network showing the metabolites connected to four microRNAs found to be involved in tamoxifen resistance. Metabolites in green are downregulated in carbohydrate-fed patients, while metabolites in red are upregulated. MicroRNAs are colored purple. **d** IPA Function plot showing metabolites involved in organismal growth. Orange arrows indicate activation, while blue arrows indicate inhibition

In the Pathway analyses, MetaboAnalyst and IPA showed complimentary information. Quantitative metabolite set enrichment analysis (MSEA) identified biologically meaningful patterns in serum metabolite concentration changes (Fig. 6a and Table 12 in Appendix). Significantly enriched pathways included energy associated metabolic pathways (amino sugar metabolism and pyruvate metabolism which links to glutamate metabolism, the citric acid cycle, gluconeogenesis and the Warburg effect). IPA showed the main functions of the involved metabolites as cellular growth and proliferation, molecular transport, small molecule biochemistry, carbohydrate metabolism and amino acid metabolism (Fig. 6b). Interestingly, the metabolites showed a pattern congruent with growth of organism (Fig. 6c) with metabolites increased in carbohydrate-fed patients activating growth pathways, and downregulation of metabolites acting as inhibitors of growth. Finally, four (miR-26a-5p, miR-30c-5p, miR126-3p and miR-210-3p) out of the seven microRNAs found to be involved in resistance to tamoxifen in our previous review [27] could indirectly be associated with the metabolic network through insulin signaling pathways (Fig. 6d). The same metabolic pathways were evident when only ER positive patients were considered.

## Discussion

We present the first study to examine the effect of per-oral preoperative carbohydrate load on perioperative metabolism in operable breast cancer patients. Among the 15 different serum metabolites that distinguished fasting from the per-oral carbohydrate load, we observed increased systemic lactate and pyruvate, decreased ketone bodies, increased glycerol, and reduced amino acids in the patients who received the carbohydrate load. Moreover, we found highly significant positive correlations between S-insulin and S-lactate and S-pyruvate. Thus, changes in these 15 key metabolites are consistent with increased glycolysis, increased ketolytic activity, reduced lipolysis, and reduced proteolysis, which are exactly the same metabolic modifications seen after carbohydrate challenge in healthy persons [28]. Being able to capture these well-known metabolic effects of insulin increases the reliability of our model to detect other changes that may follow a carbohydrate load.

It may be considered that 18 h is too short to expect effect of the carbohydrate load on tumor cell proliferation and metabolism. However, in vitro studies show that glucose fed MCF-7 cells increase their proliferation after 12–24 h [3] .Others found the same pattern in three different breast cancer cell lines [29]. As the cell lines lack the in vivo endocrine response to glucose the increased proliferation was based on GTP-ase driven phosphorylation of EGFR with increased activity and longevity of this receptor as a consequence. Also, animals fed with a diet containing increased glucose show an increased epithelial mesenchymal transition (EMT) [30].

The increased S-lactate and S-pyruvate in the carbohydrate patients stems primarily from two sources. Firstly, lactate is the product of glycolysis, especially in muscle cells, and is transported to the liver for conversion back to glucose, known as the Cori cycle [31]. The intended effect of preOP is to contra act and reduce insulin resistance that follows surgical stress [32]. This stressor leads to reduced mitochondrial ATP production and lactate formation [33, 34]. In healthy individuals, an oral glucose tolerance test (OGTT) showed a negative correlation between differences in S-glucose concentrations and differences in S-lactate levels (i.e. a rise in S-glucose leads to a reduction in S-lactate) [35]. Moreover, during 180 min after an OGTT among non-insulin dependent diabetic mellitus (NIDDM) patients there was no significant alteration in S-lactate levels [36]. Thus, it is unlikely that preOp itself creates a systemic lactate production. Therefore, S-lactate in our patients may come from excretion of intracellular lactate and pyruvate produced in the breast cancer cells. Consequently, lactate and pyruvate in the present study are probably translocated into the systemic circulation via mono carboxylate transporter type 4 (MCT-4), which is a known part of the Warburg effect [13]. Despite the fact that systemic metabolite concentrations are functional read outs of the numerous homeostatic reactions in the body, which will blur the contribution from the cancer cell metabolism to the serum levels [18], our present observation of positive correlation between larger tumor size and increasing S-lactate is supported by Hui S et al. [37].. Also, the positive correlation between proliferation and tumor size solely occurs in the carbohydrate group this suggests that carbohydrate exposure to larger tumors (i.e.T2 tumors) increases both proliferation and S-lactate. Thus, this indicates that lactate from the Warburg effect in the tumor cells may have a substantial contribution to the systemic lactate and pyruvate levels. This observation also adheres to the lack of correlation between intra tumor lactate/pyruvate and fasting/carbohydrate status in the present study, as the former are probably excreted from the cells into the systemic environment.

Moreover, tumor cells not only produce lactate for excretion through MCT-4, they are also able to take up systemic circulating lactate and pyruvate via the MCT-1 transporters [38]. Regardless of the source, systemic lactate and pyruvate will certainly benefit the free CTCs shed from the tumor during surgery that are on their way to distant tissue to form micrometastases [39] but may also benefit the preoperatively established occult micrometastases [40, 41]. Lactate and pyruvate are the most preferred substrates for lactate/pyruvate dehydrogenase (LDH/PDH), ensuring a 1:1 ratio between lactate and pyruvate when equilibrium is reached. Thus, LDH provides substrate for both the production of ATP via the tricarboxylic acid (TCA) cycle [37] and also increased gluconeogenesis for the production of ribose for nucleotide synthesis via the pentose phosphate pathway (PPP) [38]. Notably, increased levels of serum LDH [42] and increased expression of LDH in breast cancer tissue [43] and lung tumors [44] are associated with an inferior prognosis.

In line with other studies [45], we observed a positive correlation between higher proliferation and increased glutamate content in tumor tissue. Glutamate is a metabolic product of glutaminolysis, which drives membrane trafficking to promote breast cancer cell invasiveness [46]. In addition, the expression of glutaminase genes *GLS* and *GLS2* correlates with increased tumor growth rates [47]. Many tumors become glutamine-dependent, as it serves as a direct route into the TCA cycle at the alpha-ketoglutaric acid level with consequential ATP production. Together with glycine and cysteine, glutamate is a precursor to the tripeptide glutathione, which is an antioxidant molecule that serves to ‘buffer’ superoxide insults encountered in the tumor microenvironment [45]. Glutathione is the major thiol-containing endogenous antioxidant and serves as a redox buffer against various sources of oxidative stress. In tumors, maintaining a supply of glutathione is critical for cellular survival because it allows cells to resist the oxidative stress associated with rapid metabolism, DNA-damaging agents, and inflammation, among others [48, 49]. Glucose metabolism and biosynthesis of glutathione are often modulated by the PI3K/Akt pathway, which is often dysregulated in breast cancer tumors [50, 51]. Importantly, one of the effects of targeting the PI3K/Akt-pathway upstream [52] and downstream [19] is reduced glutathione content in tumor cells. In the PPP-pathway, NAD+ and NADP are converted into NADH and NADPH, respectively, which contribute to maintaining glutathione (GSSG) in the reduced state (GSH) [53]. Thus, the PPP-pathway in the Warburg effect secures a high intracellular level of glutathione, which is regarded as the most important cellular protection system against attack from reactive oxygen species (ROS) in both dividing and hibernating luminal cells [10], and also in cancer stem cells [54]. Thus, preoperative carbohydrate loading seems to create a doubly favorable environment that will probably serve the CTCs liberated during surgery [39] more than the already established micrometastases [55]. First, CTCs have a surplus of cellular fuel via lactate and pyruvate available systemically. Second, they benefit from an increased level of intracellular protection systems against ROS via increased tumor glutathione. Both effects will increase the probability of CTCs thriving and surviving as micrometastases, which then may erupt as clinical relapse years later, compatible with the tumor biology of luminal breast cancers. However, our observed clinical endpoint between 3 to 7 years must be regarded as ‘early relapses’ when coming to luminal cancers [56]. Thus, we need a much longer follow up to capture the late recurrences in order to get the correct picture of the clinical outcome of the present study.

Several attempts have been made to reverse the above-mentioned metabolic pathways for treatment purposes. The first attempt was to reverse the Warburg effect with the polyphenol resveratrol*,* which blocks PDH/LDH. In colon cancer cells, resveratrol inhibits proliferation, gluconeogenesis, and PPP [57]. By blocking PDH, resveratrol promotes mitochondrial electron transport chain overload with increased ROS production, ultimately resulting in apoptosis [58]. Secondly, a ketogenic diet has been shown to be effective in preclinical studies [59]. A ketogenic diet produces a large amount of intracellular ketone bodies that have a direct cytotoxic effect. Furthermore, the ketogenic state inhibits insulin/IGF signaling and downstream signaling pathways, such as PI3K/Akt/mTOR [60]. Interestingly, in the present study, the patients in the fasting group reached a ketogenic state with increased ketone bodies, which may have created an unfavorable environment for the cancer cells in the tumor and for the liberated CTCs. This is in line with a recent RCT of using ketogenic diet as adjuvant treatment in one of the study arms. They observed a better overall survival in the group that received ketogenic diet [61]. Others have recently shown a profound effect of ketogenic diet in a xenografted breast cancer mouse model with increased ketone bodies and increased aminoacidic [62], which is in line with our observations. The authors hypothesize that the anti-cancer effect may be mediated through immunological mechanisms [62]. Thus, use of a ketogenic diet as adjuvants to conventional therapy is rooted in several studies [63].

Likewise, physical activity is known to prevent and improve survival in several cancer forms and is thus recommended as a measure to both prevent and treat breast cancer [64, 65]. One of the mechanisms behind these observation is a change in the estrogen metabolism after 180 min exercise pr. week. They found an increased 2 hydroxy-estrone level known to antagonize the estradiol action [66] This observation is important for both in the preventive setting as breast cancer risk is correlated to total life exposure of estrogens [67]. Also, changes in diet affect the cancer incidence [68], and also prognosis in breast cancer patients [69].

A combination of calorie restriction and physical exercise in postmenopausal women did also reduce insulin levels [70]. In our patients, we found that metabolic changes after the carbohydrate load affected the ER-positive breast cancer patients. Thus, ketogenic diet combined with physical exercise would probably be beneficial for our patients as this approach will affect both the ER and insulin signaling pathways.

Interestingly, intermittent fasting (i.e. caloric restriction for 16–48 h [71] has been proven to affect the metabolism and disease process in a beneficial manner. Notably, intermittent fasting in animal studies have demonstrated reduction of tumor size [72]. In humans, intermittent fasting improves insulin sensitivity and thus reduces insulin and IGF-1 related signaling in over weighted individuals [72, 73]. Preclinical studies show that intermittent fasting more than 2 days is as effective as chemotherapy to reduce cancer load [74]. Thus, the ketones derived from intermittent fasting decreases cancer cell viability by attacking several hallmarks of cancer [75].

The IPA-analyses confirmed that the systemic response to the carbohydrate load converge towards pathways involved in proliferation and growth of the organism. Moreover, other pathways related to the Warburg effect were also involved. Thus, peroral preoperative carbohydrate load shifts the systemic metabolism towards a very fortunate and beneficial environment for CTCs liberated from the tumor under the operation. Interestingly, four out of seven microRNAs related to endocrine resistance [27] also regulate the same metabolic pathways through insulin signaling pathways, which are known to be involved in endocrine resistance with reduced effect of tamoxifen and aromatase inhibitors. Thus, it seems plausible to introduce metformin early on as adjuvant treatment to regain the endocrine sensitivity. Intriguingly, circulating microRNAs from the tumor in exosomes [76] can perform cell-independent microRNA biogenesis and promote tumorigenesis away from the primary tumor [77]. Thus, we may speculate that one of the steps in the metastatic process is to control the systemic metabolic pathways to ensure a beneficial environment and survival of the liberated cancer cells [54]. Moreover, increased cellular uptake of glucose via the Warburg effect [10] favor differentiating glycosylation of intracellular proteins included paucimannosylation [78]. Intriguingly, the metastatic Epithelial-Mesenchymal-Transition (EMT) process is regulated through glycosylation of key regulator proteins, that are frequently modulated via the insulin /IGF signaling [79]. Thus, glycosylation opens up a connection between the glucose/insulin signaling and increased survival of CTCs trough enhancement of the EMT-processes.

Taken together, this explorative study indicates that the carbohydrate loading state and fasting state have opposite systemic and micro-environmental effects, which may explain why the relapses in the present study were skewed towards the carbohydrate group, with an inferior RFS, BCSS, and OS in patients with high tissue glutathione, high S-lactate, and high S-pyruvate. The favorable macro- and micro-environmental changes for the tumor that come from carbohydrate loading reflect the Warburg effect, which serves the CTCs and micrometastases more than the patient [80]. In luminal cancers, the Warburg pathway enzyme PFKFB4 acts as a molecular fulcrum that couples sugar metabolism to transcriptional activation by stimulating the ER co-activator SRC-3 to promote aggressive metastatic tumors [81].

The present study has several weak points. First, it is a post hoc explorative analysis of an RCT. Therefore, the various analyses are not sufficiently powered regarding the various endpoints. In addition, tissue samples were not available for all patients, which reduces the number of patients in the various analyses. Thus, this creates a greater risk of a type II error than a type I error. Furthermore, the tissue analyses were skewed towards patients with larger tumors. This could introduce systematic error in the analysis. However, tumor size was not included in the final Cox models in any survival analysis, indicating that this error was not strong enough to blur the effects of the metabolites. Also, including diet recalls and demographic data of the patients would have strengthened the study. Detecting the well-known endocrine metabolic fingerprint of insulin strengthens the method and the reliability of the various findings in this study. However, the study is too small to conclude on preoperative preparation guidelines; fasting or carbohydrate loading. Moreover, the pilot nature of the present study calls for validation in a larger study with a long-term follow-up. Introducing a ketogenic diet as a third study arm may test out whether ketone bodies could wipe out the liberated CTCs and thus improve survival.

## Conclusion

Preoperative oral glucose loading increases systemic levels of lactate and pyruvate, and tumor levels of glutathione and glutamate in luminal breast cancer patients. In fasting patients, the proapoptotic ketone bodies are increased. These biological changes may contribute to the survival differences observed between these two study groups. Integrated Pathway Analysis (IPA) in serum revealed activation of five major anabolic metabolic networks contributing to proliferation and growth mainly through insulin signaling pathways.

## Data Availability

The data that support the findings of this study are available from Stavanger Breast Cancer Research Group, but restrictions apply to the availability of these data, which were used under license for the current study and as such are not publicly available. However, data are available from the authors upon reasonable request and with permission from Stavanger Breast Cancer Research Group.

## Appendix

**Table 8:** ROC – analysis with ‘Relapse / No relapse’ as dichotomous variable in ER+ patients

Test variable	AUC	95% CI	Sensitivity (%)	Specificity (%)	*P*	Threshold
S-lactate	0.769	0.609 – 0.929	57	88	0.024	56.9
S-pyruvate	0.765	0.541 – 0.989	71	86	0.026	12.5
Tumor-Glutathione	0.711	0.485 – 0.938	100	66	0.260	1.09
S-preoperative Insulin	0.724	0.554– 0.896	86	67	0.059	18.3 I.U./L
S-preoperative insulin c-peptide	0.735	0.566 – 0.903	86	67	0.049	1.22 nM

**Table 9:** Metabolites correlated to serum insulin C peptide (Pearson’s correlation) for all patients, carbohydrate group, and fasting groups

Metabolite	*R* All	*P* All^a^	*R* CH	*P* CH^a^	*R* F	*P* F^b^
Lactate	**0.611**	**<0.001**	0.401	0.047	**0.577**	**<0.001**
Pyruvate	**0.596**	**<0.001**	0.395	0.051	**0.431**	**0.010**
Acetate	**-0.513**	**<0.001**	-0.344	0.092	-0.092	0.598
N-acetylgroups	**-0.398**	**0.002**	-0.092	0.663	**0.397**	**0.018**
Valine	**-0.366**	**0.004**	0.189	0.366	**0.385**	**0.023**
Acetoacetate	**-0.352**	**0.006**	-0.058	0.781	0.348	**0.041**
Isoleucine	**-0.333**	**0.009**	-0.166	0.428	**0.488**	**0.003**
Lysine	-0.301	**0.020**	-0.064	0.763	**0.646**	**<0.001**
Propylene Glycol	-0.260	**0.045**	0.044	0.833	0.305	0.075
Citrate	0.244	0.060	0.262	0.205	0.348	**0.041**
Leucine	-0.242	0.063	0.125	0.551	**0.416**	**0.013**
Glucose	0.233	0.074	0.345	0.092	0.187	0.282
Creatine	-0.209	0.110	-0.444	0.026	0.005	0.978
Phenylalanine	-0.206	0.115	0.357	0.080	**0.503**	**0.002**
Methanol	-0.185	0.157	-0.162	0.438	-0.269	0.118
Glycerol	-0.152	0.246	-0.111	0.596	0.323	0.059
Glutamine	-0.152	0.248	0.216	0.300	0.058	0.740
Alanine	0.150	0.252	0.207	0.320	0.303	0.077
Isopropyl alcohol	-0.134	0.307	0.185	0.375	0.222	0.199
Threonine	-0.108	0.409	0.000	1.000	**0.462**	**0.005**
3-Hydroxybutyrate	-0.098	0.455	0.153	0.464	**0.439**	**0.008**
Creatinine	-0.077	0.557	0.176	0.399	0.158	0.365
Dimethylsulfone	0.076	0.565	0.264	0.202	0.332	0.051
Acetone	0.027	0.835	0.078	0.710	**0.530**	**0.001**
Proline	-0.014	0.913	0.154	0.462	0.049	0.778
Asparagine	-0.012	0.927	-0.126	0.547	**0.624**	**<0.001**
Glycoproteins	0.012	0.928	0.003	0.989	**0.606**	**<0.001**
Methionine	0.010	0.938	0.412	0.041	0.138	0.430

^a^Significant at *p* ≤ 0.045 after Benjamini-Hochberg correction

^b^Significant at *p* ≤ 0.041 after Benjamini-Hochberg correction

**Table 10:** Metabolites correlated to serum Insulin Growth Factor Binding Protein 3 (IGFBP3) (Pearson’s correlation) for all patients, carbohydrate group, and fasting groups

Metabolite	*R* (All)	*P* (All)*	*R* (CH)	*P* (CH)*	*R* (F)	*P* (F)*
Isoleucine	**0.424**	**0.001**	0.414	0.040	0.351	**0.039**
Glycoproteins	**0.410**	**0.001**	0.224	0.282	0.478	**0.004**
Asparagine	**0.401**	**0.001**	0.364	0.073	0.399	**0.018**
Leucine	**0.393**	**0.002**	0.107	0.612	0.440	**0.008**
Acetone	**0.383**	**0.003**	0.298	0.148	0.397	**0.018**
Lysine	**0.378**	**0.003**	-0.017	0.937	0.459	**0.006**
N.acetylgroups	**0.342**	**0.007**	-0.241	0.247	0.484	**0.003**
Phenylalanine	**0.322**	**0.012**	0.114	0.586	0.314	0.066
Propylene-Glycol	**0.321**	**0.012**	0.044	0.833	0.349	**0.040**
Isopropyl-alcohol	**0.319**	**0.013**	-0.079	0.706	0.434	**0.009**
Alanine	**0.310**	**0.016**	0.100	0.635	0.413	**0.014**
Acetoacetate	0.243	**0.062**	0.036	0.866	0.192	0.268
Threonine	0.206	0.114	-0.250	0.228	0.395	**0.019**
Valine	0.196	0.134	0.050	0.814	0.082	0.640
Acetate	0.180	0.169	0.079	0.707	0.066	0.707
Lactate	-0.166	0.205	-0.347	0.090	0.207	0.232
pyruvate	-0.164	0.211	-0.287	0.164	0.131	0.453
Methionine	-0.158	0.228	-0.484	0.014	-0.066	0.704
Glycerol	0.137	0.296	-0.384	0.058	0.360	**0.034**
Proline	-0.124	0.345	-0.358	0.079	0.004	0.982
Creatine	0.103	0.435	0.175	0.401	0.051	0.773
Creatinine	0.084	0.522	-0.189	0.365	0.146	0.403
Methanol	0.079	0.549	0.288	0.162	-0.110	0.528
Glutamine	0.075	0.569	-0.176	0.399	0.123	0.481
Glucose	0.055	0.678	0.007	0.972	0.186	0.285
Citrate	0.031	0.812	-0.052	0.804	0.134	0.442
Dimethylsulfone	-0.005	0.967	-0.255	0.219	0.096	0.584
3-Hydroxybutyrate	-0.001	0.993	-0.320	0.119	0.046	0.794

*Abbreviations*: *CH* Carbohydrate group, *F* Fasting group, *P* Pearson’s correlation *p*-value, *R* Pearson’s correlation *R* value.

*Significant at *p* ≤ 0.01 after Benjamini-Hochberg correction for multiple testing

**Significant at *p* ≤ 0.037 after Benjamini-Hochberg correction for multiple testing

***Significant at *p* ≤ 0.04 after Benjamini-Hochberg correction for multiple testing

**Table 11:** Metabolites correlated to serum Insulin Growth Factor 1 (IGF1) (Pearson’s correlation) for all patients, carbohydrate group, and fasting groups

Metabolite	*R* (All)	*P* (All)^a^	*R* (CH)	*P* (CH)^a^	*R* (F)	*P* (F)^a^
Methionine	-0.318	0.013	-0.591	**0.002**	-0.135	0.438
Isopropyl_alcohol	-0.314	0.015	-0.318	0.121	-0.330	0.052
Creatinine	-0.302	0.019	-0.381	0.061	-0.265	0.124
Proline	-0.274	0.034	-0.465	0.019	-0.095	0.585
Valine	-0.251	0.053	-0.122	0.563	-0.406	0.015
Propylene_Glycol	-0.230	0.077	-0.159	0.446	-0.291	0.089
Acetoacetate	-0.230	0.077	-0.168	0.421	-0.308	0.072
Methanol	-0.218	0.094	-0.001	0.996	-0.400	0.017
Acetone	-0.184	0.159	-0.061	0.772	-0.247	0.152
pyruvate	-0.173	0.186	-0.271	0.191	-0.179	0.303
Leucine	-0.160	0.222	-0.090	0.670	-0.208	0.231
3.Hydroxybutyrate	-0.157	0.230	-0.231	0.266	-0.114	0.515
Dimethylsulfone	0.155	0.238	0.032	0.880	0.245	0.155
Threonine	-0.152	0.248	-0.397	0.050	0.013	0.941
Lactate	-0.139	0.289	-0.323	0.115	-0.026	0.882
N.acetylgroups	-0.131	0.320	-0.385	0.057	-0.038	0.830
Glycerol	-0.125	0.342	-0.430	0.032	0.075	0.668
Lysine	-0.118	0.370	-0.169	0.418	-0.104	0.551
Glutamine	0.100	0.447	-0.053	0.801	0.257	0.136
Isoleucine	-0.096	0.468	0.116	0.582	-0.225	0.193
Acetate	-0.092	0.483	0.031	0.883	-0.148	0.395
Creatine	-0.087	0.507	-0.002	0.994	-0.154	0.378
Glycoproteins	-0.081	0.539	-0.059	0.779	-0.088	0.614
Citrate	0.063	0.631	0.115	0.584	0.014	0.936
Alanine	-0.048	0.717	-0.070	0.738	-0.048	0.783
Glucose	0.017	0.899	-0.008	0.969	0.079	0.652
Phenylalanine	0.011	0.931	0.253	0.222	-0.080	0.648
Asparagine	-0.006	0.962	0.393	0.052	-0.163	0.349

*Abbreviations*: *CH* Carbohydrate group. *F* Fasting group, *P* Pearson’s correlation *p*-value, *R* Pearson’s correlation *R* value.

^a^Significant at *p* ≤ 0.002 after Benjamini-Hochberg correction for multiple testing

**Table 12:** Results from Quantitative Metabolite Set Enrichment Analysis

Metabolic pathway	Total Cmpd	Hits	Statistic Q (Expected 1.613)	Raw p	FDR
Amino Sugar Metabolism	33	3	24.50	0.000	0.000
Propanoate Metabolism	42	1	44.09	0.000	0.000
Valine, Leucine and Isoleucine Degradation	60	4	31.60	0.000	0.000
Pyruvate Metabolism	48	3	28.66	0.000	0.000
Phenylalanine and Tyrosine Metabolism	28	2	31.54	0.000	0.000
Fatty Acid Biosynthesis	35	3	25.58	0.000	0.000
Aspartate Metabolism	35	3	16.23	0.000	0.000
Ethanol Degradation	19	1	33.64	0.000	0.000
Tyrosine Metabolism	72	1	32.22	0.000	0.000
Butyrate Metabolism	19	1	32.22	0.000	0.000
Lysine Degradation	30	1	31.52	0.000	0.000
Biotin Metabolism	8	1	31.52	0.000	0.000
Carnitine Synthesis	22	1	31.52	0.000	0.000
Ammonia Recycling	32	3	14.09	0.000	0.000
Warburg Effect	58	5	13.16	0.000	0.000
Cysteine Metabolism	26	1	27.23	0.000	0.000
Pyruvaldehyde Degradation	10	1	27.23	0.000	0.000
Urea Cycle	29	3	13.38	0.000	0.000
Glutamate Metabolism	49	3	13.38	0.000	0.000
Gluconeogenesis	35	3	17.45	0.000	0.000
Ketone Body Metabolism	13	2	17.24	0.000	0.000
Glycolysis	25	2	13.61	0.000	0.000
Citric Acid Cycle	32	2	14.00	0.000	0.001
Glycine and Serine Metabolism	59	5	8.26	0.000	0.001
Alanine Metabolism	17	2	13.75	0.000	0.001
Transfer of Acetyl Groups into Mitochondria	22	3	9.34	0.001	0.001
Glucose-Alanine Cycle	13	3	9.17	0.001	0.002
Pyrimidine Metabolism	59	1	12.64	0.005	0.008
Nicotinate and Nicotinamide Metabolism	37	1	12.64	0.005	0.008
Purine Metabolism	74	1	12.64	0.005	0.008
Phenylacetate Metabolism	9	1	12.64	0.005	0.008
Threonine and 2-Oxobutanoate Degradation	20	1	7.42	0.035	0.048
Methionine Metabolism	43	1	6.38	0.052	0.065
Betaine Metabolism	21	1	6.38	0.052	0.065
Spermidine and Spermine Biosynthesis	18	1	6.38	0.052	0.065
Glycerolipid Metabolism	25	1	5.74	0.065	0.080
Galactose Metabolism	38	2	2.87	0.184	0.219
Arginine and Proline Metabolism	53	2	0.94	0.575	0.665
Glutathione Metabolism	21	1	0.27	0.692	0.743
Selenoamino Acid Metabolism	28	1	0.27	0.692	0.743
Tryptophan Metabolism	60	1	0.27	0.692	0.743
Sphingolipid Metabolism	40	1	0.00	0.969	0.969
Lactose Synthesis	20	1	0.00	0.969	0.969
Lactose Degradation	9	1	0.00	0.969	0.969

## Abbreviations

ATP: Adenosine triphosphate
AUC: Area under the curve
BCSS: Breast cancer specific survival
CI: Confidence interval
CPMG: Carr–Purcell–Meiboom–Gill spectra
CTC: Circulating tumor cell
EMT: Epithelial mesenchymal transition
ER: Estrogen receptor
ERAS: Enhanced recovery after surgery
GSH: Glutathione, reduced form
GSSG: Glutathione, oxidized form
HER2: Human epithelial growth factor receptor 2
HR: Hazard ratio
HR-MAS-MR: High-resolution magic angle spinning - magnetic resonance
IGF1: Insulin-like growth factor 1
IGF1R: Insulin-like growth factor 1 receptor
IGFBP3: Insulin-like growth factor 1 binding protein 3
IHC: Immunohistochemistry
IPA: Ingenuity Pathway Analysis
IR: Insulin receptor
LDH: Lactate dehydrogenase
MAI: Mitotic activity index
MCT-1: Monocarboxylate transporter 1
MCT-4: Monocarboxylate transporter 4
MR: Magnetic resonance
MRI: Magnetic resonance imaging
MSEA: Metabolite Set Enrichment Analysis
NAD: Nicotinamide adenine dinucleotide
NADP: Nicotinamide adenine dinucleotide phosphate
NOESY: One-dimensional ^1^H Nuclear Overhauser effect spectroscopy
NSD: Norwegian Center for Research Data
OS: Overall survival
PHD: Pyruvate dehydrogenase
PLS: Partial least square
PLS-DA: Partial least square discriminant analysis
PPH3: Phosphorylated phosphohistone 3
PPP: Pentose phosphate pathway
PR: Progesterone receptor
RCT: Randomized controlled trial
RFS: Relapse free survival
ROS: Reactive oxygen species
S: Serum
SRC-3: Steroid receptor co-activator 3
TCA-cycle: Tri carboxyl acid cycle
